# Biomarkers of connective tissue disease‐associated interstitial lung disease in bronchoalveolar lavage fluid: A label‐free mass spectrometry‐based relative quantification study

**DOI:** 10.1002/jcla.24367

**Published:** 2022-03-25

**Authors:** Jing Ye, Pengcheng Liu, Renming Li, Hui Liu, Wenjing Pei, Changxiu Ma, Bing Shen, Dahai Zhao, Xiaoyu Chen

**Affiliations:** ^1^ Department of Pulmonary and Critical Care Medicine The Second Affiliated Hospital of Anhui Medical University Hefei China; ^2^ 12485 School of Basic Medicine Anhui Medical University Hefei China

**Keywords:** BALF, CTD‐ILD, LC‐MC/MS, pathogenesis, proteomics

## Abstract

**Background:**

The pathogenesis of connective tissue disease‐associated interstitial lung disease (CTD‐ILD) is unclear. This study aims to identify differentially expressed proteins (DEPs) in CTD‐ILD to determine the potential role of these DEPs that may play in the pathogenesis of CTD‐ILD and to offer potential therapeutic targets.

**Methods:**

Bronchoalveolar lavage fluid (BALF) samples were collected from four patients with CTD‐ILD and four patients without CTD‐ILD. Label‐free mass spectrometry‐based relative quantification was used to identify the DEPs. Bioinformatics were used to determine the potential biological processes and signaling pathways associated with these DEPs.

**Results:**

We found 65 upregulated DEPs including SFTPD, CADM1, ACSL4, TSTD1, CD163, LUM, SIGLEC1, CPB2, TGFBI and HGD, and 67 downregulated DEPs including SGSH, WIPF1, SIL1, RAB20, OAS3, GMPR2, PLBD1, DNAJC3, RNASET2 and OAS2. The results of GO functional annotation for the DEPs showed that the DEPS were mainly enriched in the binding, cellular anatomical entity, cellular processes, and biological regulation GO terms. The results of KEGG analyses showed that the pathways most annotated with the DEPs were complement and coagulation cascades, metabolic pathways, pathways in cancer, and PPAR signaling pathway. COG analyses further informed the functions associated with these DEPs, with most focused on signal transduction mechanisms; posttranslational modification, protein turnover, chaperones; intracellular trafficking, secretion, and vesicular transport; amino acid transport and metabolism; and lipid transport and metabolism.

**Conclusions:**

DEPs identified between patients with vs. without CTD‐ILD may play important roles in the development of CTD‐ILD and are potential new biomarkers for early diagnosis of CTD‐ILD.

## Graphical abstract

Differentially expressed proteins (DEPs) in bronchoalveolar lavage fluid of connective tissue disease‐associated interstitial lung disease (CTD‐ILD) patients were determined by label‐free LC‐MS/MS method. We found 65 upregulated and 67 downregulated DEPs, which may play important roles in the development of CTD‐ILD and are potential new biomarkers for early diagnosis of CTD‐ILD.

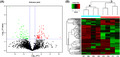

## 1 INTRODUCTION

Interstitial lung disease (ILD) is the most common complication associated with connective tissue diseases (CTDs), and most of these patients have poor outcomes. Nearly 15% of patients with CTD have secondary ILD, and approximately 15% of patients with ILD have potential CTD.^1^ The most common CTDs involving the lung and associated with ILD include scleroderma, mixed CTD, systemic lupus erythematosus, dermatomyositis/polymyositis, Sjögren's syndrome, and rheumatoid arthritis. The median survival time for patients with CTD‐associated ILD (CTD‐ILD) is approximately 6.5 years. The number of individuals with CTD who die of ILD is 123.6 per 1000 person‐years.^2^, ^3^ CTD‐ILD is usually characterized by occult progressive dyspnea and intermittent cough, with symptoms often obscured by extrapulmonary manifestations such as arthritis or muscle weakness. Without effective treatment, patients will experience respiratory failure.

The etiology of CTD‐ILD is unclear. At present, environmental pathogens are hypothesized to be a cause of pathogenic inflammation. Environmental pathogens may cause inflammatory cells to invade interstitial and alveolar spaces, eventually leading to damage of the alveolar epithelial. The extent of eventual recovery of the lung structure and function is likely determined by two factors: the intensity of the process and the level of disruption of the normal lung extracellular matrix, especially the layers that define the alveolar structure.^4^, ^5^ Another hypothesis for the pathogenic inflammation is that some CTD subtypes occur with lung injury, causing local inflammation and inducing autoantigen expression, which leads to the production of autoantibodies in the lung. This may continue through a subsequent combination of disease‐related autoantibodies and antoantigens, resulting in fibrosis and lung inflammation.^6^ Therefore, it is urgent to determine the underlying pathological mechanisms and find new therapeutic targets for CTD‐ILD.

Next‐generation transcriptome sequencing and highly sensitive mass spectrometry (MS) instrumentation have been advancing in recent years, improving both genomic and proteomic technologies. Therefore, studies are increasingly being conducted to reevaluate correlations between disease states and gene or protein expression using recently generated data sets. In proteomics, evaluating a mixture of proteins using liquid chromatography‐mass spectrometry/mass spectrometry (LC‐MS/MS), sometimes called shotgun proteomics, is still the first choice for large‐scale protein identification. For protein quantification, a relative quantitative strategy based on label‐free MS is becoming an increasingly favored alternative to the label‐based method.^7^ The two major approaches for the relative quantification of proteins using label‐free MS are data‐independent acquisition strategies and data‐dependent acquisition experiments.

Bronchoalveolar lavage fluid (BALF) was extracted from the lungs with a bronchoscope. The biochemical components of BALF include mainly proteins and phospholipids, followed by nucleic acids (e.g., mRNA, DNA, and miRNA). These components mirror the pathophysiological state of the patients; therefore, they are considered rich sources of biomarkers, with some biomarkers established for use in clinical applications.^8^

In the present study, we used label‐free LC‐MS/MS analyses to identify the protein composition and their relative levels in BALF specimens obtained from patients with CTD‐ILD and control patients with community‐acquired pneumonia (CAP). Using bioinformatics analyses, we aimed to identify potential biomarkers of CTD‐ILD and the key signaling pathways involved in the occurrence of CTD‐ILD.

## 2 MATERIALS & METHODS

### 2.1 Experimental design

**FIGURE 1:**
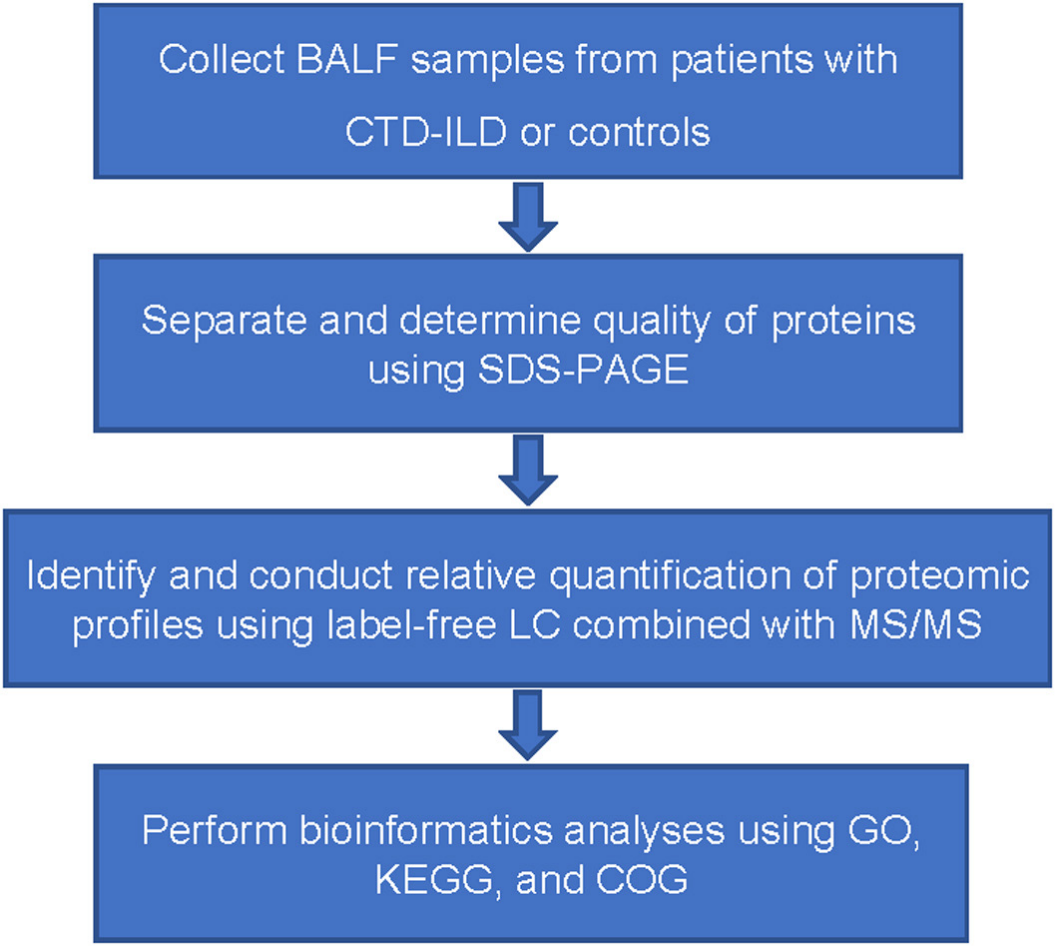
Flowchart of the experimental design. BALF represents bronchoalveolar lavage fluid; CTD‐ILD, connective tissue disease‐associated interstitial lung disease; COG, Clusters of Orthologous Groups; GO, Gene Ontology; KEGG, Kyoto Encyclopedia of Genes and Genomes; LC, liquid chromatography; MS/MS, tandem mass spectrometry; and SDS‐PAGE, sodium dodecyl sulfate‐polyacrylamide gel electrophoresis

The study design is shown in the flowchart in Figure 1.

### 2.2 Patients and diagnoses

From August to September 2020, BALF samples were collected from eight patients who had not received hormone or immunosuppressant treatment and had been admitted to the Second Affiliated Hospital of Anhui Medical University. The samples were collected from four patients with CTD‐ILD (experimental group) after their diagnosis but before hormone or immunosuppressant treatment, and from four patients with ‐CAP but without immune system disease (control group; samples obtained from the healthy lung side). The clinical diagnosis and some sociodemographic characteristics for each of the eight patients are given in Table 1.

**TABLE 1:** Clinical diagnosis and sociodemographic characteristics of eight participants

Case No.	Diagnosis	Sex	Age (years)	Group
Case 1	CTD‐ILD	Female	52	E1
Case 2	CTD‐ILD	Female	76	E2
Case 3	CTD‐ILD	Female	72	E3
Case 4	CTD‐ILD	Female	49	E4
Case 5	CAP	Male	59	C1
Case 6	CAP	Male	43	C2
Case 7	CAP	Male	55	C3
Case 8	CAP	Female	22	C4

Abbreviations: C, control group; CAP, community‐acquired pneumonia; CTD‐ILD, connective tissue disease‐associated Interstitial lung disease; E, experimental group.

The ILD diagnosis was based on the guidelines described in chapter 92 of Goldman's Cecil Medicine, 24th Edition.^9^ The diagnostic criteria for CTD‐ILD were based on the recommendations of the American College of Rheumatology and the American Rheumatism Association.^10^, ^11^, ^12^, ^13^ In the present study, all patients were examined by respiratory specialists and rheumatologists.

The study was approved by the Medical Ethics Committee of the Second Affiliated Hospital of Anhui Medical University (approval number YX2020‐106), and all participants provided written informed consent in a manner consistent with the Declaration of Helsinki.

### 2.3 BAL procedure and immediate sample processing

Bronchoalveolar lavage (BAL) is a part of a routine diagnostic examination. BAL was performed according to the European Respiratory Society guidelines.^14^ In brief, patients were restricted from drinking and fasted for 6 h before the procedure. Midazolam and lidocaine (2%) were used for local upper airway anesthesia. Bronchofiberoscopy was performed using an Olympus BF‑H190 bronchoscope (Olympus). A sterile isotonic sodium chloride solution at 37°C was dripped into the right middle or left lung lingual side in equal portions of 20 ml through a sterile syringe. The total lavage volume was 4 ml/kg of body weight. The fluid was immediately aspirated by gentle suction after each aliquot. If the recovery volume exceeded 50% of the drip volume, it was considered a representative sample. The sample was processed immediately after BAL. The supernatant was absorbed and centrifuged at 3000 × *g* for 10 min, and stored at −80°C until it was used in the experiments.

### 2.4 BALF protein extraction

Each BALF sample for analysis was diluted 1:4 with a trichloroacetic acid solution and mixed well. The samples were precipitated at 4°C overnight. The intermixture was centrifuged at 15,000 × *g* at 4°C for 15 min. The supernatant was discarded. We then added pre‐cooled acetone solution to precipitate the samples for 30 min, centrifuged the samples at 15,000 × *g* at 4°C for 15 min, and removed the supernatant. This step was repeated another time. After the resulting precipitate was dried for 5 min, protein lysate (AP0601‐50; Beijing Bangfei Biotechnology Company) was added, and the samples were incubated overnight at 4°C. The protein solution was obtained after being centrifuged at 10,000 × *g* at 4°C for 3 min.

The total protein in each BALF sample without protein degradation was resolved by sodium dodecyl sulfate‐polyacrylamide gel electrophoresis (SDS‐PAGE). Coomassie blue staining showed clear, complete, and uniform protein bands.

### 2.5 Enzymatic hydrolysis of protein

A dithiothreitol solution (1 M; 5 µl) was added to 60 μg of protein solution, and the mixture was kept at 37°C for 1 h. Iodoacetamide solution (20 µl) was added, and the mixture remained at room temperature for 1 h in the dark. The samples were centrifuged, and the supernatant was discarded. We added 100 µl of uric acid buffer (8 M urea, 100 mM Tris‐HCl, pH 8.0) to the precipitate, centrifuged the samples, and discarded the supernatant. This step was repeated twice. The samples were treated with NH_4_HCO_3_ (50 mM, 100 µl) and centrifuged, and the supernatant was discarded. Trypsin was added to the protein in a ratio of 1:50, and enzyme digestion was proceeded at 37°C for 12–16 h.

### 2.6 LC‐MS/MS analysis

Using an LC‐MS/MS system, we identified the differentially expressed proteins (DEPs) between patients with or without CTD‐ILD.^15^ Each BALF sample was separated using a high‐performance liquid chromatography system with nanoliter flow rates. The chromatographic column was equilibrated with 95% of mobile phase A. Mobile phase A was 0.1% formic acid in the water, and mobile phase B was 0.1% formic acid in acetonitrile. The phase B gradient was linearly increased at 0–2 min to 6%–9%; 2–10 min to 13%; 10–50 min to 26%, 50–70 min to 38%; and 70–71 min to 100%; and maintained at 100% for 71–78 min. Each sample was separated by capillary high‐performance liquid chromatography and analyzed by label‐free MS with an Orbitrap Fusion Tribrid mass spectrometer (Thermo Scientific).

### 2.7 Sequence database search and data analysis

Proteome Discoverer (version 2.4; Thermo Fisher Scientific) software was used for data analysis. Peptide identification was performed with the SEQUEST search engine using human proteome databases containing reviewed Uniprot sequences downloaded from Uniprot. Decoys for the database search were generated with the reverse function. The relative quantification levels of proteins in both replicates of experimental samples significantly different from control samples (*p* values <0.05) and with fold changes (FCs) ≥1.5 were considered upregulated proteins, whereas those with FC ≤0.667 were considered downregulated proteins. A FC between 0.667 and 1.5 or with *p* > 0.05 indicated no change in protein expression between the two groups.^16^, ^17^, ^18^

### 2.8 Gene Ontology (GO), Kyoto Encyclopedia of Genes and Genomes (KEGG), and Cluster of Orthologous Groups of Proteins (COGs) signaling pathway analyses

We performed GO functional annotation analysis on the identified DEPs, which were mapped to terms in the GO database (http://www.geneontology.org/) to calculate the number of proteins per term. The hypergeometric test was used to identify GO entries significantly enriched among the DEPs compared with the background proteins. We performed KEGG functional annotation to identify the biochemical metabolic pathways and signal transduction pathways significantly enriched among the identified DEPs. The COGs were obtained by comparing the sequence of the proteome with the COG database. Protein functional descriptions and classifications were obtained.

### 2.9 Enzyme linked immunosorbent assay (ELISA)

ELISA was performed according to the instructions (Elabscience Biotechnology Co., Ltd). After the process for BALF samples, optical density (OD) value was measured at a wavelength of 450 nm using a microplate reader. The protein concentrations of BALF samples were calculated using a standard curve. All experiments were repeated for three times.

### 2.10 Statistical analysis

GraphPad Prism software (version 5.0, San Diego, California) was used to analyze the experimental results of Mann‐Whitney tests (two‐tailed). Fisher's chi‐square test was used to compare the categorical variable (only sex). Values are expressed as means ± SEM, and *p* < 0.05 was considered statistically significant.

## 3 RESULTS

### 3.1 Participants

There was no significant difference detected between the participants in the experimental group and control group for age (mean ± SEM, 62.3 ± 6.9 vs. 44.8 ± 8.3 years; *n* = 8, *p* = 0.16) or sex (4 women in the experimental group vs. 3 men and 1 woman in the control group: *p* = 0.92).

### 3.2 SDS‐PAGE

**FIGURE 2:**
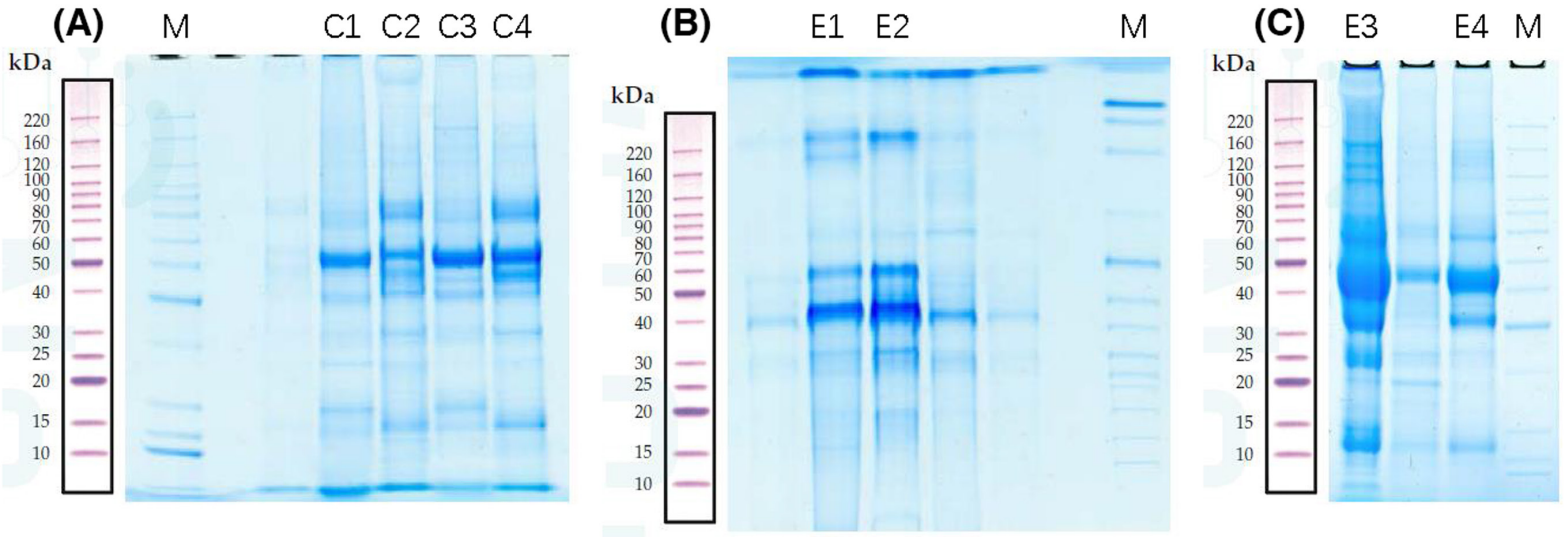
Sodium dodecyl sulfate‐polyacrylamide gel electrophoresis. In this representative western blot, M is the marker. C1–C4 are high‐abundance proteins obtained from bronchoalveolar lavage fluid (BALF) samples from patients 1–4 in the control group; and E1–E4 are high‐abundance proteins obtained from BALF samples from patients 1–4 in the experimental group

BALF samples obtained from four patients in the experimental group and 4 patients in the control group were each separated using SDS‐PAGE. In the molecular weight range of 15–220 kDa, total proteins from the eight samples were effectively separated without protein degradation. The protein levels were sufficient for subsequent experiments (Figure 2).

### 3.3 LC‐MS/MS analysis and identification of DEPs

**FIGURE 3:**
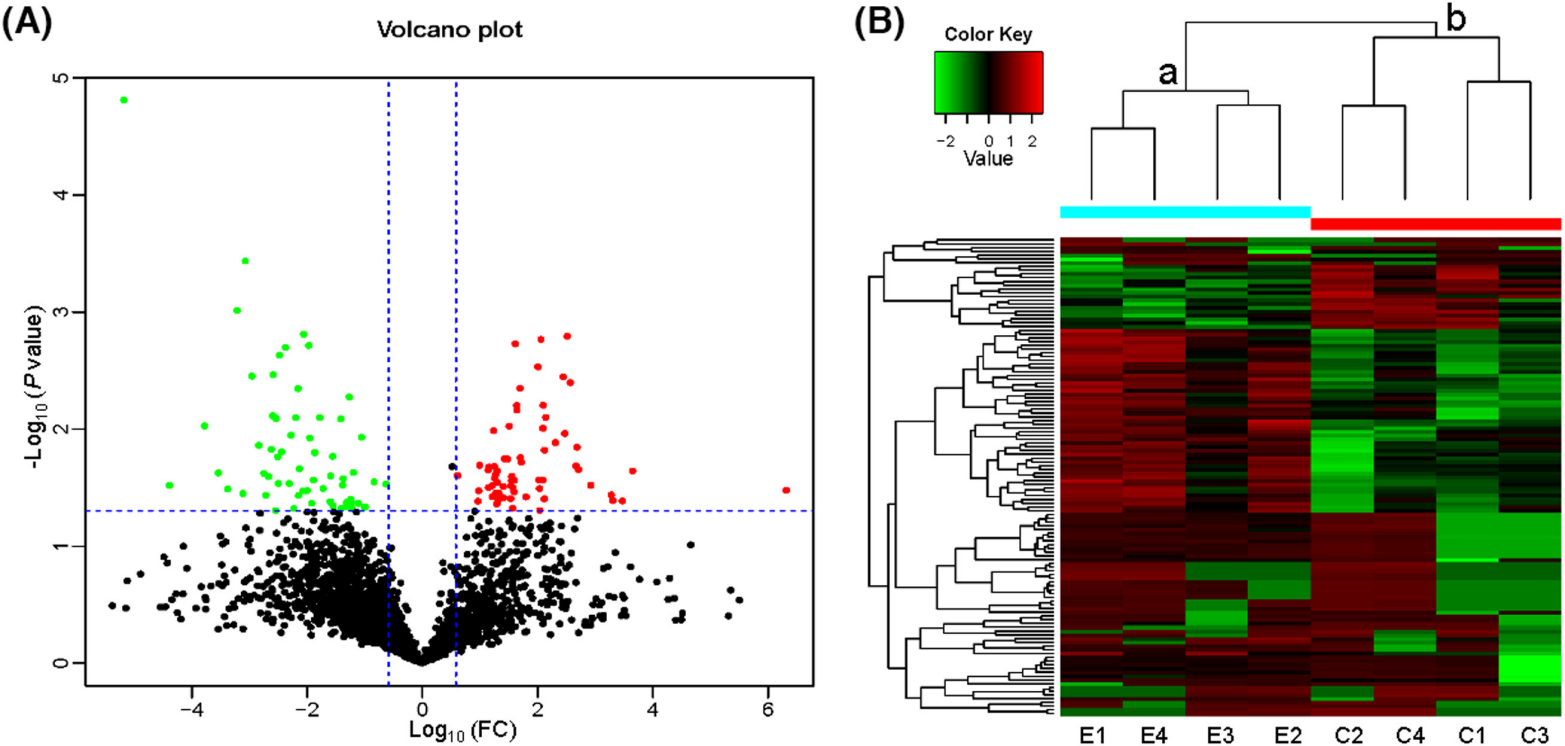
Volcano plot and heat map of differentially expressed proteins (DEPs) in patients with or without CTD‐ILD. (A) In this volcano plot, red dots represent proteins with a significant fold change (FC) ≥1.5 and/or *p* < 0.05; green dots, proteins with a significant FC ≤0.667; and black dots, no obvious changes in proteins. (B) In the heat map, the upregulated and downregulated DEPs are observed by cluster analysis. Each row in the figure represents a protein, each column is a sample (C1–C4 indicates control group patients 1–4; E1–E4, experimental group patients 1–4), and the colors represent different expression levels (the log10 values of the quantitative value are obtained and median correction is performed during the heat map drawing). These results indicate that there is a significant difference in protein levels expressed in the bronchoalveolar lavage fluid of the patients with or without CTD‐ILD

LC‐MS/MS was used to identify and provide relative quantification of proteins in samples of BALF from each participant. In total, 3835 proteins were identified in the BALF of patients with or without CTD‐ILD. Of 127 DEPs detected, 65 were upregulated and 67 were downregulated. Table 2 showed the top 10 upregulated and downregulated proteins. We plotted the magnitude of the FC (log10 FC) to obtain a volcano plot (Figure 3A). The cluster analysis of the DEP expression showed that the expression patterns in patients with CTD‐ILD differed from those in patients without CTD‐ILD, and the protein expression within each group was clustered together (Figure 3B).

**TABLE 2:** Analysis of differential expressed proteins

Regulated	Accession	Gene name	Description	FC	*p* value
UP	P35247	SFTPD	Pulmonary surfactant‐associated protein D	80.005	0.033
UP	Q9BY67	CADM1	Cell adhesion molecule 1	12.598	0.023
UP	O60488	ACSL4	Long‐chain‐fatty‐acid‐‐CoA ligase 4	11.124	0.041
UP	Q8NFU3	TSTD1	Thiosulfate: glutathione sulfurtransferase	9.932	0.041
UP	Q86VB7	CD163	Scavenger receptor cysteine‐rich type 1 protein	9.730	0.037
UP	P51884	LUM	Lumican	7.619	0.030
UP	Q9BZZ2	SIGLEC1	Sialoadhesin	6.565	0.022
UP	Q96IY4	CPB2	Carboxypeptidase B2	6.456	0.014
UP	Q15582	TGFBI	Transforming growth factor‐beta‐induced protein ig‐h3	6.374	0.021
UP	Q93099	HGD	Homogentisate 1,2‐dioxygenase	5.956	0.004
DOWN	P29728	OAS2	2'−5'‐oligoadenylate synthase 2	0.140	0.014
DOWN	O00584	RNASET2	Ribonuclease T2	0.129	0.004
DOWN	Q13217	DNAJC3	DnaJ homolog subfamily C member 3	0.119	0.000
DOWN	Q6P4A8	PLBD1	Phospholipase B‐like 1	0.116	0.036
DOWN	Q9P2T1	GMPR2	GMP reductase 2	0.108	0.001
DOWN	Q9Y6K5	OAS3	2'−5'‐oligoadenylate synthase 3	0.965	0.032
DOWN	Q9NX57	RAB20	Ras‐related protein Rab−20	0.086	0.024
DOWN	Q9H173	SIL1	Nucleotide‐exchange factor SIL1	0.073	0.009
DOWN	O43516	WIPF1	WAS/WASL‐interacting protein family member 1	0.048	0.030
DOWN	P51688	SGSH	N‐sulphoglucosamine sulphohydrolase	0.028	0.000

Note

Accession indicates the characteristic numbers of different proteins. Description represents a detailed description of the proteins.

Abbreviation: FC, fold change.

### 3.4 GO functional annotation and enrichment analysis

**FIGURE 4:**
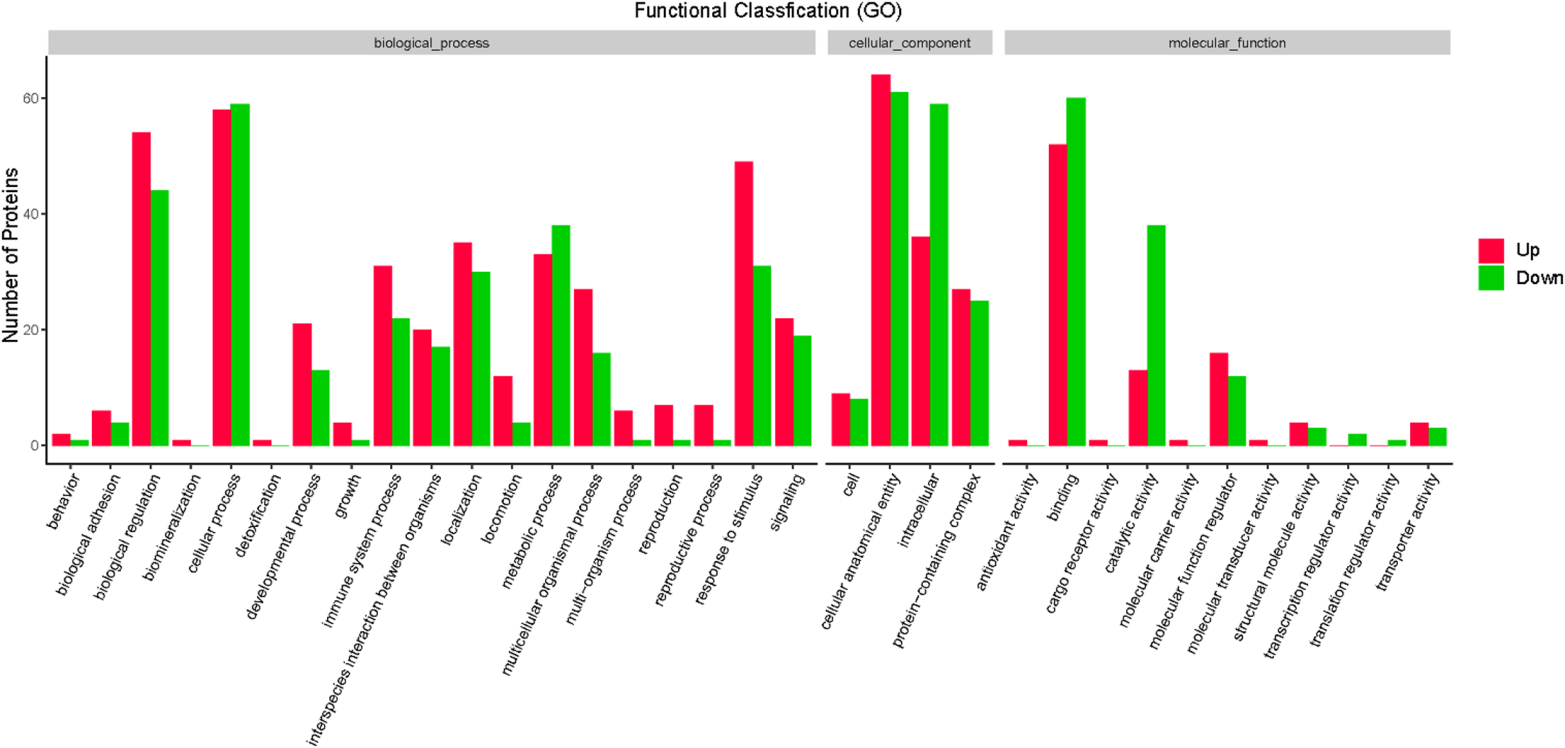
Gene Ontology (GO) annotation for functional classification of proteins differentially expressed between patients with vs. without CTD‐ILD. Up represents proteins that are upregulated between patients with vs. without CTD‐ILD, whereas down represents proteins that are downregulated

**FIGURE 5:**
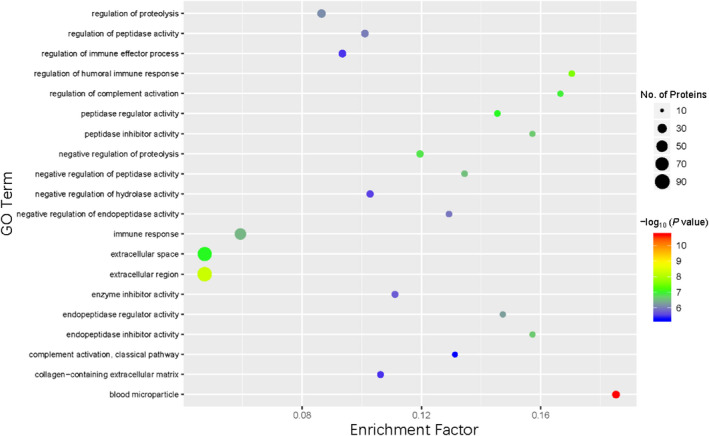
Gene Ontology (GO) enriched functional terms. The size of the circle represents the number of differentially expressed proteins in the GO term. The color of the circle indicates the *p* value for the degree of enrichment. In this scale, red represents a smaller *p* value and a higher enrichment degree. The Fisher exact test *p* value is the *p* value of the enrichment test obtained using the Fisher exact test; and −log10 (*p* value) is the log conversion of the Fisher exact test *p* value

GO analyses provides information for three functional domains—the biological processes in which the proteins participate, the cellular locations where the proteins are present, and the molecular functional roles that the gene products play—and organizes these functional concepts as a directed acyclic graph. GO enrichment provides not only candidate sets for differential protein screening but also the functional enrichment of foreground gene sets and potential functions of the proteins. Therefore, GO enrichment results increase the reliability of research focused on determining pathogenesis. Figure 4 shows the results of GO functional annotation of the DEPs between patients with or without CTD‐ILD. For the GO domain of cellular component, 64 DEPs were primarily associated with the cellular anatomical entity term. For the GO domain of molecular function, 52 DEPs were mainly associated with binding. For the biological process GO domain, the DEPs were primarily involved in biological regulation (54 DEPs) and cellular process (58 DEPs). Across all three domains, the top three upregulated proteins were surfactant protein D (SFTPD), vascular cell adhesion molecule 1 (VCAM‐1), and acyl‐CoA synthetase long‐chain family member 4 (FACL4). We further analyzed the significance of the enriched GO terms in the DEPs between patients with or without CTD‐ILD and found that the DEPs played important roles as cellular anatomical entities and in cellular processes, binding, and biological regulation (Figure 5).

### 3.5 KEGG metabolic pathway analysis

**FIGURE 6:**
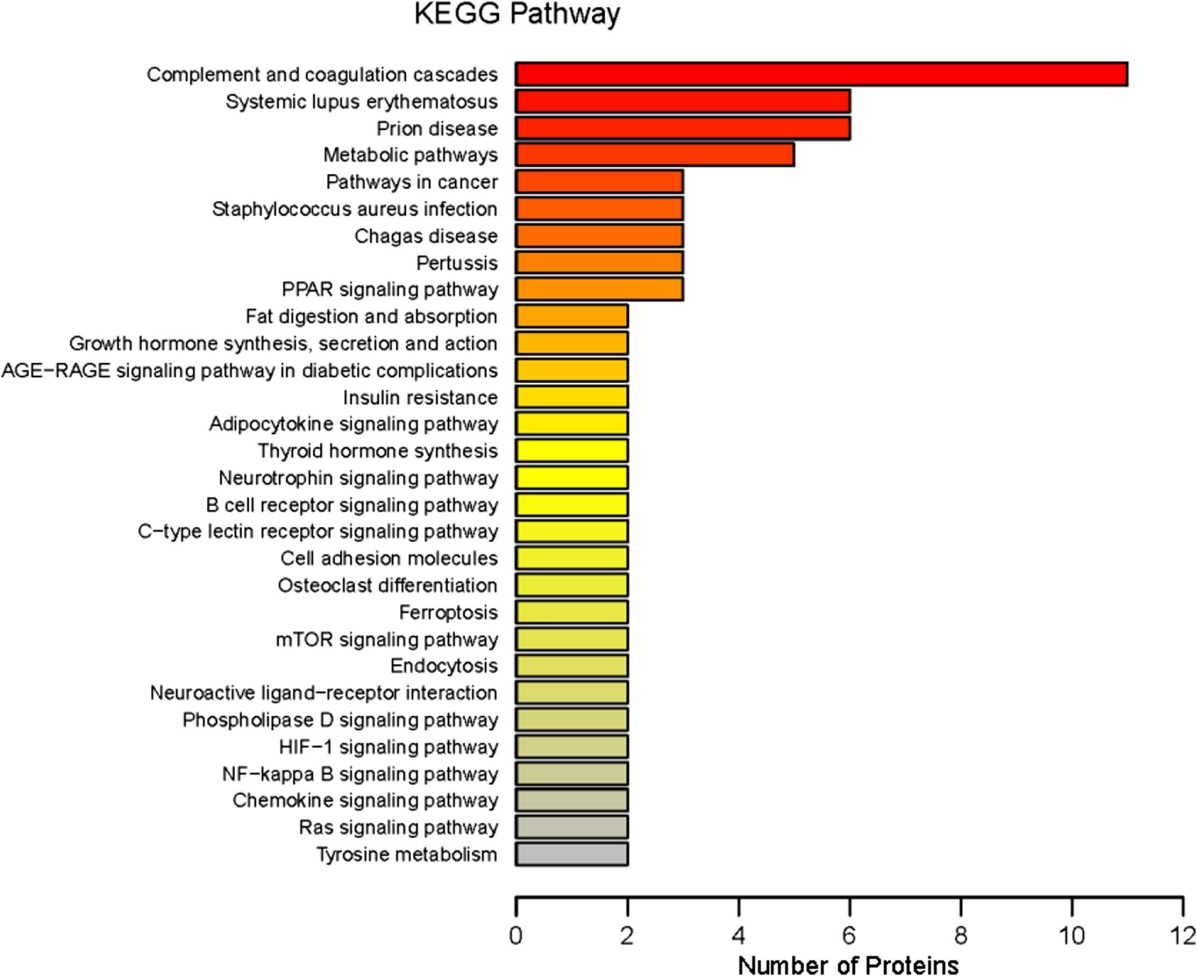
Kyoto Encyclopedia of Genes and Genomes (KEGG) pathway annotation

**FIGURE 7:**
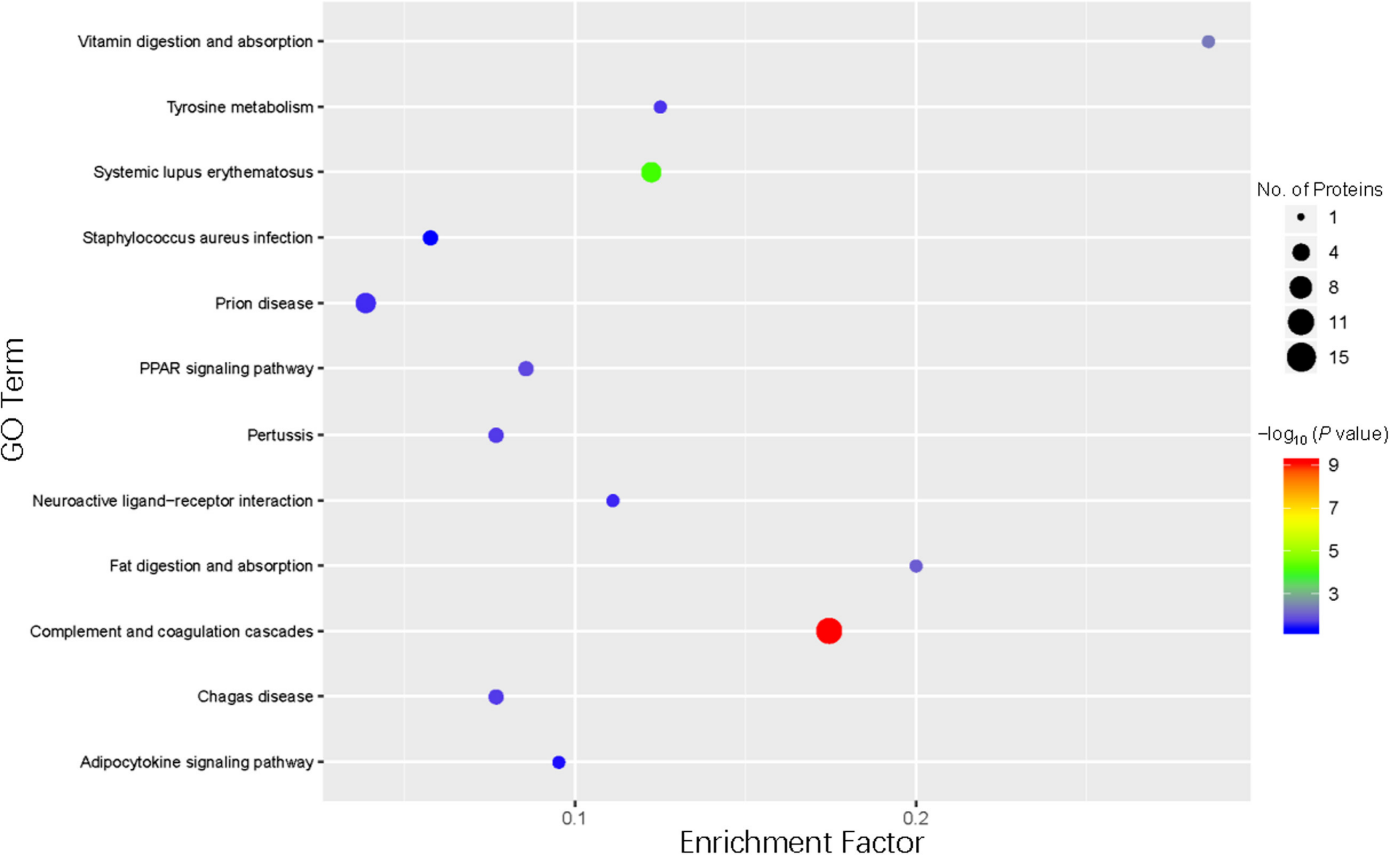
Kyoto Encyclopedia of Genes and Genomes (KEGG) pathway enrichment. The color of the circle represents the statistical significance of the finding. The Fisher exact test *p* value is the *p* value of the enrichment test obtained using the Fisher exact test; −log10 (*p* value) is the log conversion of the Fisher exact test *p* value

KEGG is a database integrating genome, chemistry, and system function information to provide a genetic and chemical blueprint. The most important signal transduction pathways and biochemical metabolic pathways associated with the genes of the DEPs can be determined using KEGG analysis. KEGG database analysis was used to assess the DEPs in BALF samples between patients with or without CTD‐ILD. The results showed that KEGG pathways annotated with the DEPs included complement and coagulation cascades, metabolic pathways, pathways in cancer, and the peroxisome proliferator‐activated receptor (PPAR) signaling pathway (Figure 6). The upregulated DEPs were complement and coagulation cascades, including CPB2, complement C5, and complement components C7 and C8γ. The upregulated DEPs associated with metabolic pathways were FACL4, HGO, MAOB, and PTGDS. The upregulated DEPs associated with pathways in cancer were IKBKB, AGT, and PLCG2. The upregulated DEPs associated with the PPAR signaling pathway were FACL4, Apo A‐I, and PLIN2. In the pathway enrichment analysis, the KEGG pathway was used as the unit, and the hypergeometric test was used to identify the significantly enriched pathways of the DEPs compared with all identified proteins. We used pathway enrichment analysis to determine the most important signal transduction pathways and biochemical metabolic pathways of DEPs (**Figure** **7**). Most of the detected upregulated proteins were focused in metabolic pathways, such as the complement and coagulation cascades pathways, the PPAR signaling pathway, and the adipocytokine signaling pathway.

### 3.6 COG protein functional analysis

**FIGURE 8:**
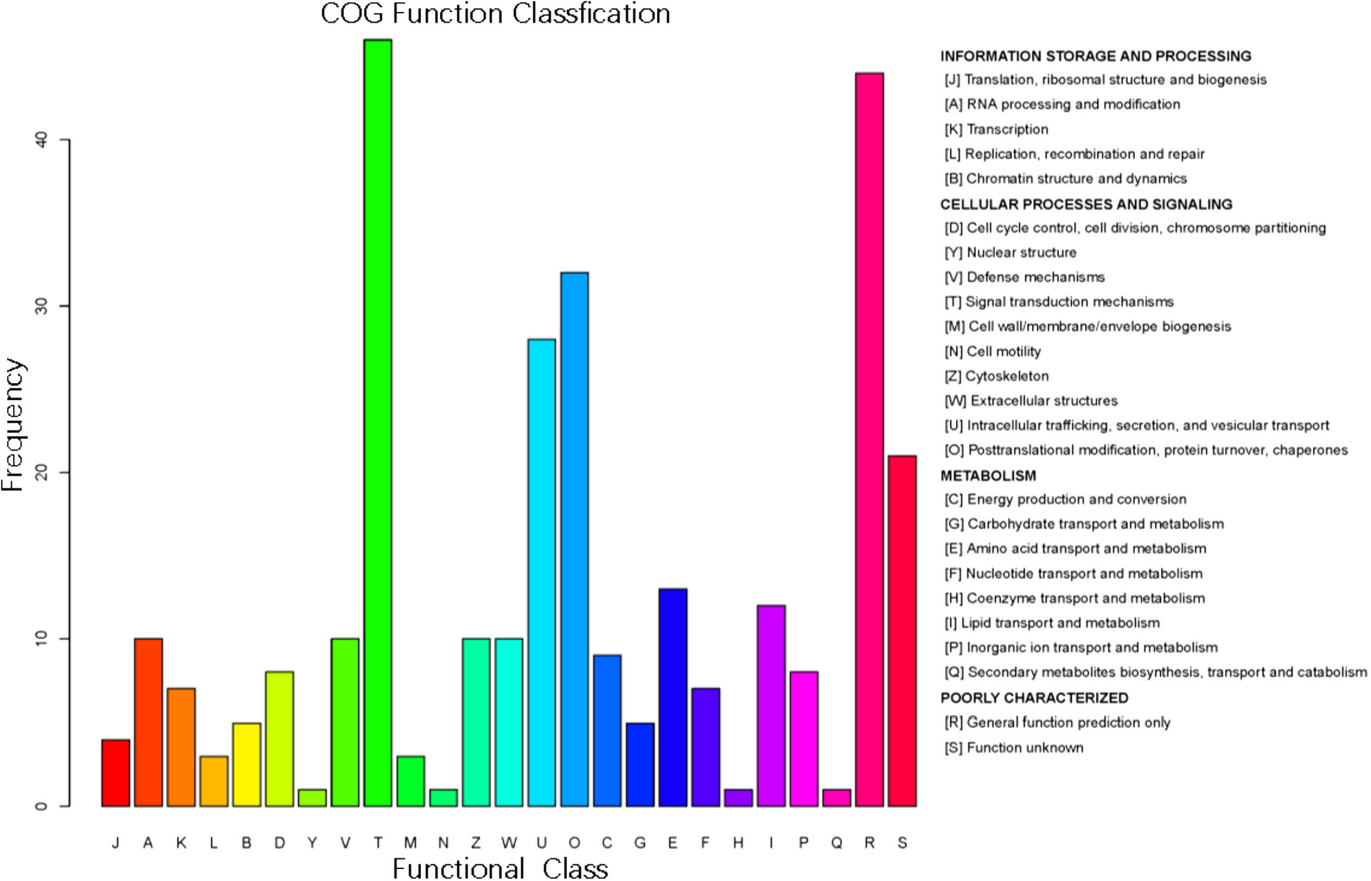
Clusters of Orthologous Groups (COGs) annotated for the differentially expressed proteins between patients with vs. without CTD‐ILD

The COG protein database is designed to enable classification of proteins from completely sequenced genomes based on phylogenic lineages.^19^ It is useful for predicting the function of a single protein and the functions of proteins in newly sequenced genomes. By using the web‐based COGNITOR program, a protein can be compared with proteins in the COG database, and it can be classified into appropriate clusters. The COG database provides retrieval and query of COG classification data. By using COG database analyses, we predicted the potential functions of the DEPs between patients with or without CTD‐ILD and performed functional classification statistics (**Figure** **8**). The results showed that the functions of these DEPs were mainly focused in signal transduction mechanisms; posttranslational modification, protein turnover, chaperones; intracellular trafficking, secretion, and vesicular transport; amino acid transport and metabolism; and lipid transport and metabolism.

### 3.7 Concentration of top DEPs in BALF

**FIGURE 9:**
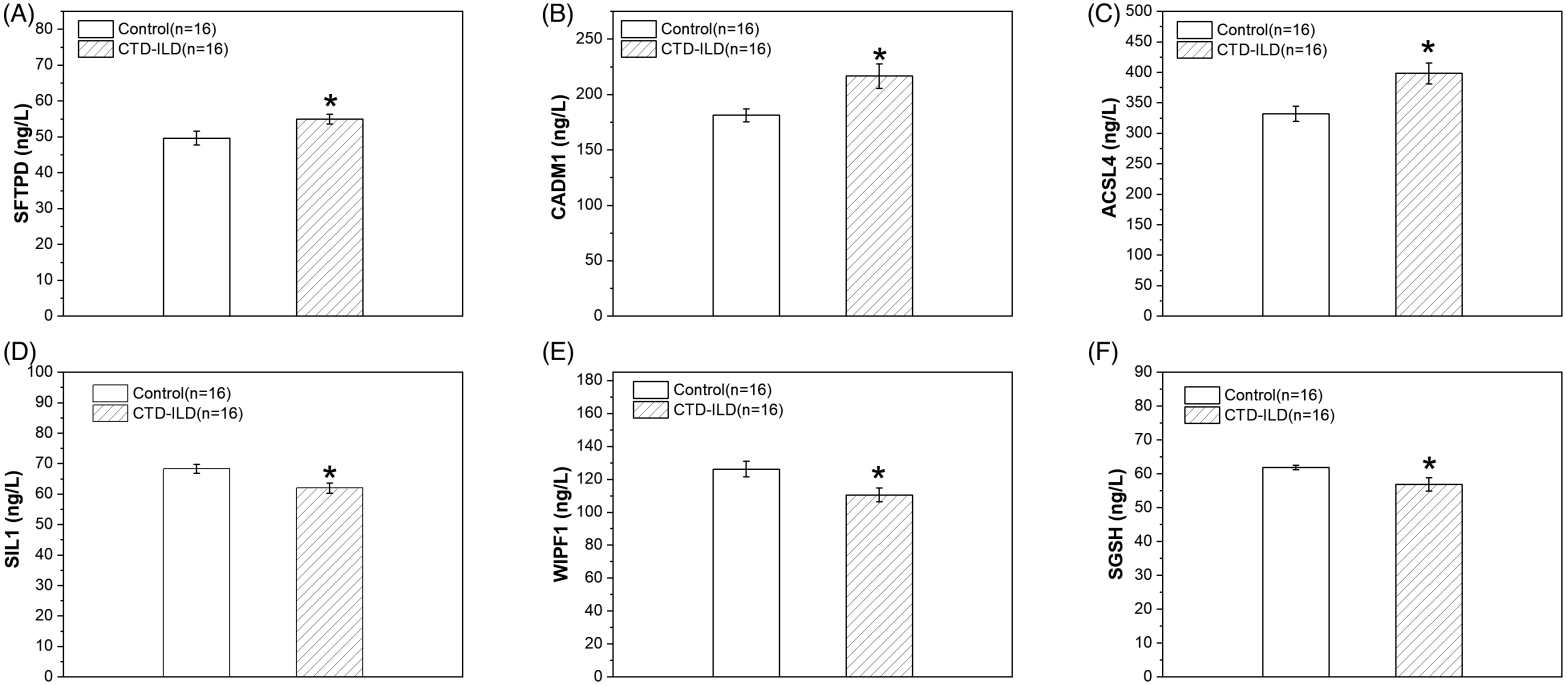
Protein concentrations of top differentially expressed proteins (DEPs) in bronchoalveolar lavage fluid (BALF). (A–F) Summary data showing the concentrations (ng/L) of pulmonary surfactant‐associated protein D (SFTPD, A), cell adhesion molecule 1 (CADM1, B), long‐chain‐fatty‐acid‐‐CoA ligase 4 (ACSL4, C), nucleotide‐exchange factor SIL1 (SIL1, D), WAS/WASL‐interacting protein family member 1 (WIPF1, E), and N‐sulphoglucosamine sulphohydrolase (SGSH, F) in BALF samples from CTD‐ILD patients and controls. Among of them, SFTPD, CADM1 and ACSL4 are upregulated DEPs, but SIL1, WIPF1 and SGSH are downregulated DEPs. Values are shown as the mean ± SEM (*n* = 16); ∗*p* < 0.05 for Control vs. CTD‐ILD

To confirm the DEPs in the LC‐MS/MS experiment, we measured top three upregulated and downregulated DEPs using ELISA experiment. Our data showed that the expressions of SFTPD, CADM1 and ACSL4 were significantly upregulated in BALF samples of CTD‐ILD group compared to control group. On the contrary, the expressions of SIL1, WIPF1 and SGSH were significantly downregulated in BALF samples of CTD‐ILD group compared to control group (Table 3 and Figure 9). Therefore, these results are consistent with the data from our LC‐MS/MS experiment.

**TABLE 3:** Mean concentration of top three upregulated and downregulated differential expressed proteins

Regulated	Gene name	Mean concentration (Mean ± SE, ng/L)	
		Control	CTD‐ILD
UP	SFTPD	49.63 ± 1.94	54.96 ± 1.38*
UP	CADM1	181.37 ± 5.83	216.65 ± 43.62*
UP	ACSL4	331.99 ± 12.68	398.34 ± 17.32*
DOWN	SIL1	68.37 ± 1.47	61.97 ± 1.64*
DOWN	WIPF1	126.23 ± 4.73	110.53 ± 4.14*
DOWN	SGSH	61.87 ± 0.64	56.88 ± 2.02*

Note

*p < 0.05.

## 4 DISCUSSION

Patients with CTD often experience secondary ILD, a significant cause of death among patients with CTD. However, it may be challenging to distinguish ILD from other pulmonary disorders, especially when symptoms of ILD present before other obvious signs of CTD, potentially delaying critical treatment for these patients. At present, the pathological mechanisms underlying CTD‐ILD are still unclear. Proteomics may be useful in the discovery not only of new information regarding the pathogenesis of CTD‐ILD but also of biomarkers related to this disease. Given the continued development of MS and bioinformatics, it is possible to identify a large number of proteins in a sample simultaneously. In the present study, we used label‐free LC‐MS/MS to identify 3835 proteins in the BALF of patients with or without CTD‐ILD and found 127 DEPs in patients with CTD‐ILD. Among of these DEPs, 65 proteins were upregulated, and 67 proteins were downregulated. We further confirmed these finding by ELISA experiments and showed that the expression levels of SFTPD, CADM1 and ACSL4 were significantly upregulated in BALF samples in CTD‐ILD group compared to control group. On the contrary, the expression levels of SIL1, WIPF1 and SGSH were significantly downregulated in BALF samples in CTD‐ILD group compared to control group. SFTPD encodes surfactant protein D (SP‐D), which belongs to the collectin (collagen‐containing calcium‐dependent lectin) family, humoral molecules of the innate immune system that are present in both plasma and mucosal surfaces. Collectin proteins are oligonucleotide proteins consisting of carbohydrate recognition domains connected to collagenous structures that recognize molecular patterns related to pathogens.^20^ Most studies investigating SP‐D in association with CTD‐ILD have assessed patients with systemic sclerosis (SSc).^21^, ^22^ Serum SP‐D levels in patients with SSc are significantly higher than in healthy controls, and serum SP‐D levels in patients with systemic sclerosis‐associated ILD (SSc‐ILD) are significantly higher than in patients without SSc‐ILD. As an index to evaluate lung involvement, SP‐D reflects the state of pulmonary fibrosis but does not reflect the dynamic progress of pulmonary fibrosis.^23^ SP‐D has also been shown to be a diagnostic biomarker in rheumatoid arthritis‐associated ILD and in dermatomyositis/polymyositis‐ILD.^24^, ^25^ These findings are consistent with our MS results. CADM1 is a key adhesion receptor regulating the adhesion of human lung mast cell (HLMC) with primary human lung fibroblast and human airway smooth muscle cell, which is believed to be important in the progression of idiopathic pulmonary fibrosis.^26^ Therefore, we assume that there are similar manifestations in CTD‐ILD, which may become an interesting therapeutic target in future. ACSL4 is an isozyme using arachidonate as a substrate and belongs to the acyl‐CoA ligase family, also known as FACL4. The main function of FACL4 is to participate in the metabolism of free long‐chain fatty acids in cells and transform them into fatty acyl‐CoA esters. There are no previous reports of FACL4 in CTD‐ILD, and whether FACL4 is related to the pathogenesis of CTD‐ILD will require further study. SIL1 functions as a nucleotide‐exchange factor for the endoplasmic reticulum (ER) heat‐shock protein of 70 kDa (Hsp70) Bip in eukaryotic cells. SIL1 may catalyze the ADP release from Bip by interacting directly with the ATPase domain of Bip.^27^ ER stress has been linked with various respiratory diseases, including infections, cystic fibrosis, idiopathic pulmonary fibrosis and asthma.^28^ Therefore, we speculate that there may be a similar pathway in the pathological process of CTD‐ILD. The gene encoded by Wiskott–Aldrich syndrome protein interacting protein family member 1 (WIPF1) is frequently mutated in Wiskott‐Aldrich syndrome.^29^ WIPF1 was found to be a oncogene in colorectal cancer, glioma and breast cancer.^30^ However, the role of WIPF in ILD is unclear. N‐sulfoglucosamine sulfohydrolase (SGSH) is known to be involved in desulfation of glycosaminoglycan chains on proteoglycans. Glycosaminoglycans are important components in extra cellular matrix turnover in the lungs and the altered extra cellular matrix composition is an important factor in pulmonary fibrosis.^31^

Our GO analyses of the DEPs showed enrichment in all three GO domains. The top three upregulated proteins in each of these domains were identified as SFTPD, VCAM‐1, and FACL4. As mentioned above, SP‐D can not only reflect the degree of pulmonary fibrosis, but also be used as a diagnostic biomarker of some CTD‐ILD. The role of FACL4 in the occurrence and development of CTD‐ILD is still unclear, which needs further research in future. VCAM‐1, a member of the immunoglobulin superfamily, plays a key role in the development of atherosclerosis and rheumatoid arthritis. Because VCAM‐1 is expressed in large and small blood vessels after being stimulated by cytokines and it mediates the adhesion of lymphocytes, monocytes, eosinophils, and basophils to vascular endothelium, playing a role in signal transduction.^32^, ^33^ Lung specimens from patients with SSc and mainly cellular nonspecific interstitial pneumonia show increased VCAM‐1 expression.^34^ Compared with that in SSc, the expression of VCAM‐1 is more pronounced in granulomatosis with polyangiitis. High VCAM‐1 protein levels in the peripheral blood of patients with idiopathic pulmonary fibrosis can predict mortality.^35^ But the expression pattern and the assumed putative role of VCAM‐1 as a contributor to idiopathic pulmonary fibrosis pathogenesis remains unclear. It is also unclear what role of VCAM‐1 plays in the occurrence and development of CTD‐ILD.

The top 3 downregulated proteins in each of the GO domains were junctional adhesion molecule‐like (JAML), polypeptide N‐acetylgalactosaminyltransferase 1 (GALNT1), and nucleoside diphosphate kinase B (NDPKB). Junctional adhesion molecules regulate the interactions between leukocyte and endothelial and mucosal epithelial cells as well as the steady state and barrier function of epithelial cells.^36^ The gene encoding JAML, a member of the immunoglobulin superfamily, contains 2 extracellular immunoglobulin‐like domains, a transmembrane segment, and a cytoplasmic tail. JAML mRNA is expressed in hematopoietic tissues and is prominently expressed in granulocytes. JAML protein is localized on the cell plasma membrane in the contact area between cells, whereas it is not detected at the edge of free cells, which indicates that JAML is involved in hemophilic binding. JAML released from transmigrating neutrophils across inflamed epithelia may thus promote recruitment of leukocytes and aid in clearance of invading microorganisms.^37^ The expression of JAML in monocytes is significantly increased in the presence of proinflammatory factors, while the soluble extracellular recombinant of JAML significantly inhibits the adhesion and migration of monocytes mediated by proinflammatory factors. In addition, knockdown of JAML in a human acute monocytic leukemia cell line, THP‐1, decreases adhesion and migration of monocytes.^38^ Therefore, we speculate that JAML may affect the adhesion and migration of monocytes during the occurrence of CTD‐ILD.

GALNT1 is a member of a large family of Golgi resident polypeptide N‐acetylgalactosamine (GalNAc)‐transferases, and regulates mucin‐type O‐linked glycosylation, catalyzing the transfer of a‐GalNAc from UDP‐GalNAc to Ser or Thr residues of proteins. Early studies reported that GALNT1 is highly expressed in a variety of tumors, promotes tumor growth and metastasis, and is associated with poor prognosis.^39^ However, later studies showed that GALNT1‐deficient mice exhibit a bleeding disorder and lack B‐cell maturation.^40^ Whether there is a similar result associated with GALNT1 downregulation in CTD‐ILD needs additional study. Nucleoside diphosphate kinases (NDPKs) are pivotal to a variety of cellular activities, including cell proliferation, differentiation, adhesion, molecular transport, and apoptosis. NDPKB catalyzes UDP and ATP to form UTP, which is necessary to activate T helper (Th)1 and Th2 CD4^+^ T cells.^41^, ^42^ Although NDPKA and NDPKB have been associated with various cellular and biochemical functions, few of them have been confirmed in physiological relevant systems in vivo.

KEGG enrichment analysis of the DEPs in the present study showed that CTD‐ILD is associated with complement and coagulation cascades, metabolic pathways, pathways in cancer, and the PPAR signaling pathway. The upregulated DEPs associated with complement and coagulation cascades were CPB2, complement C5, and complement components C7 and C8γ. The complement cascade pathway is an enzyme cascade reaction activated by an antibody‐dependent classical pathway, antibody‐independent alternative pathway, a mannose‐binding lectin pathway, and a C5 combination pathway directly activated by thrombin.^43^ It is the first line of defense against invasive pathogens and an important part of innate immunity. There are interactions between the coagulation and complement systems. In vivo, CPB2 is the main regulator of C3a and C5a activity.^44^ Both C3a and C5a have a functionally important C‐terminal arginine, which mediates binding to their respective receptors and provides a target site for their degradation by the plasma basic carboxypeptidases, carboxypeptidase N (CPN) and CPB2.^45^ Both CPN and CPB2 may be involved in regulating the activities of these pleiotropic anaphylatoxins. Understanding the different characteristics of CPN and CPB2 in regulating the activities of these important biologically relevant peptides in different diseases may lead to the discovery of novel therapies.^46^ Some studies have shown that inhibitor of kappa light polypeptide gene enhancer in B‐cells, kinase beta (IKBKB), which is upregulated in the cancer pathway, causes human combined immunodeficiency.^47^ IKBKB is an important catalytic subunit of the IKK complex, forming the IKK complex together with catalytic subunit IKKα and regulatory subunit IKKγ. Phosphorylation of the IKK complex leads to increased activation of the NF‐κB signaling pathway to inhibit apoptosis as well as leading to T‐ and B‐cell functional defects.^48^ Therefore, we speculate that the upregulation of IKBKB detected in the BALF of patients with CTD‐ILD may be related to immune hyperfunction.

PPAR‐γ is a nuclear transcription receptor with extensive tissue and cell protection. PPAR‐γ ligands are known to inhibit TGF‐β‐induced myofibroblast differentiation by targeting the PI3K/Akt pathway in the treatment of fibrosis.^49^

Overall, our findings are consistent with previous studies from other groups. Thus, through KEGG analyses, we may provide several potential mechanisms underpinning the development of CTD‐ILD and study direction for CTD‐ILD. However, in the present study many findings are based on proteomics and bioinformatics analysis. It is hard to focus on one specific point to investigate deeply. Future experimental study should be helpful to clarify the exact molecular mechanism involving the occurrence and development of CTD‐ILD.

## 5 CONCLUSIONS

We used label‐free LC‐MS/MS technology to identify DEPs in BALF samples between patients with or without CTD‐ILD. The present study provided further supporting evidence for a role of the complement and coagulation cascades in the pathogenesis of CTD‐ILD and offered new evidence of potential biomarkers for the early diagnosis of CTD‐ILD. Our analyses of potentially involved signaling pathways also suggested many key proteins that may participate in the development of CTD‐ILD, and these proteins may be new targets for future research and treatment of CTD‐ILD.

## CONFLICT OF INTEREST

The authors report no conflicts of interest in this work.

## AUTHOR CONTRIBUTIONS

Jing Ye and Pengcheng Liu conceived and designed the experiments, performed the experiments, analyzed the data, prepared figures and/or tables, authored or reviewed drafts of the paper, and approved the final draft. Renming Li and Hui Liu were responsible for collecting and processing samples. Wenjing Pei was responsible for statistics. Changxiu Ma and Bing Shen conceived and designed the experiments, performed the experiments, authored or reviewed drafts of the paper, and approved the final draft. Dahai Zhao and Xiaoyu Chen conceived and designed the experiments, authored or reviewed drafts of the paper, and approved the final draft.
